# 单操作孔胸腔镜肺叶切除术的初步报道

**DOI:** 10.3779/j.issn.1009-3419.2010.01.03

**Published:** 2010-01-20

**Authors:** 向阳 初, 志强 薛, 连斌 张, 晓斌 侯, 克峰 马

**Affiliations:** 100853 北京，中国人民解放军总医院胸外科 Department of Toracic Surgery, PLA General Hospital, Beijing 100853, China

**Keywords:** 电视胸腔镜手术, 单操作孔, 肺叶切除术, Video-assisted thoracoscopic surgerg, Single utility port, Lobectomy

## Abstract

**背景与目的:**

电视辅助胸腔镜外科手术（video-assisted thoracoscopic surgery, VATS）已被广泛应用于胸部疾病的诊断和治疗，本研究旨在探讨单操作孔VATS肺叶切除术的可行性及临床价值。

**方法:**

2009年9月-2009年12月，我院采用单操作孔胸腔镜肺叶切除术21例，所有手术操作均在胸腔镜下完成，其中右上肺叶切除12例，左下肺叶切除5例，右下肺叶切除2例，左肺上叶切除1例，右肺中叶切除1例。

**结果:**

全部患者手术过程顺利，无中转开胸。平均手术时间（132.7±16.2）min，术中出血（110.5±24.6）mL；胸腔引流管拔出时间（3.1±1.3）d；术后住院时间（5.2±3.2）d。全部患者术后恢复顺利，无肺不张、肺部感染、出血等并发症，无围手术期死亡。

**结论:**

单操作孔胸腔镜肺叶切除术在技术上是安全、可行的，具有创伤更小、恢复更快等优点。

## Abstract (en)

**Background and objective:**

Video-assisted thoracoscopic surgery (VATS) has been widely used in the diagnosis and treatment of chest diseases, The aim of this study was to explore the feasibility and clinical value of lobectomy with single utility port complete VATS.

**Methods:**

From September 2009 to December 2009, 21 cases underwent lobectomy with single utility port complete VATS. Of 21 patients, right upper lobectomy was 12 cases, left lower lobectomy 5 cases, right lower lobectomy 2 cases, left upper lobectomy 1 case, right middle lobectomy 1 case.

**Results:**

The operation process were smooth in all patients and without conversion to thoracotomy. The mean operative time was (132.7±16.2)min and the mean intraoperative blood loss was (110.5±24.6)mL. The average chest tube drainage time was (3.1±1.3)d, and the mean hospitalization day was (5.2±3.2)d. All patients recovered smoothly and without severe complications. There were no post-operative deaths.

**Conclusion:**

Lobectomy with single utility port VATS is technically feasible and has the advantages of minimal invasive and rapid recovery.

电视辅助胸腔镜外科手术（video-assisted thoracoscopic surgery, VATS）已被广泛应用于胸部疾病的诊断和治疗，其用于肺癌的治疗在早期存在很大的争议。最近很多研究^[1, 2]^表明VATS治疗早期肺癌的5年生存率、远期生存及局部复发与常规开胸手术相似。2009版NCCN非小细胞肺癌临床实践指南认为只要不违反肿瘤治疗原则，VATS是可手术肺癌患者合理的术式选择。VATS的切口选择方法很多，通常至少需要两个操作切口^[3]^。我院自2009年9月-2009年12月，采用“单操作孔”行VATS肺叶切除术21例，效果良好，现总结报告如下：

## 1 资料与方法

### 1.1 临床资料

全组21例，男15例，女6例；年龄27岁-76岁，平均（53.8±13.5）岁。所有患者术前均行胸部增强CT、骨扫描、腹部超声、头颅CT或MRI，8患者术前行PET-CT检查，6例患者术前行CT引导下穿刺活检明确病理。21例患者中右上肺叶切除12例，左下肺叶切除5例，右下肺叶切除2例，左肺上叶切除1例，右肺中叶切除1例。其中肺癌16例（腺癌11例，鳞癌2例，细支气管肺泡癌2例，腺鳞癌1例），炎性假瘤2例，结核瘤1例，肺孤立性纤维瘤1例，肺梭形细胞血管内皮瘤1例。肺癌患者中T1N0M0 11例，T2N0M0 3例，T1N1M0 1例，T2N1M0 1例。肺内病灶直径1.0 cm-5.5 cm，平均2.4 cm。

### 1.2 手术方法

采用双腔气管插管静脉复合麻醉，侧卧位。首先取腋后线第8肋间1.5 cm切口置入30度胸腔镜，通过胸腔镜探查确定病变部位、性质及肺门解剖情况。对于合适的病例，在腋前线第3、4或第5肋间做4 cm-5 cm小切口，用乳突牵开器，将皮肤及皮下组织牵开，切断肋间肌进入胸腔，不需要置入开胸器。对于诊断不明确的周围型病变先行肺楔形切除，明确为肺癌后行肺叶切除术。对于诊断不明确，病变位于肺叶中央，无法行肺楔形切除的直接行肺叶切除。用常规器械结合内镜器械先分离肺门血管主干，对于肺动脉、肺静脉主干均用内镜切割缝合器缝合切断，对于血管分支则根据操作的便利性采用内镜切割缝合器，或用内镜打结法，双重结扎后离断。对发育不全的肺裂，用内镜切割缝合器离断。支气管均采用内镜切割缝合器切断。肺癌病例常规清扫肺门纵隔淋巴结。支气管残端及肺创面喷涂生物蛋白胶。术毕经胸腔镜孔放置胸腔闭式引流管一根。所有手术操作完全在胸腔镜下完成（图 1）。

**1:**
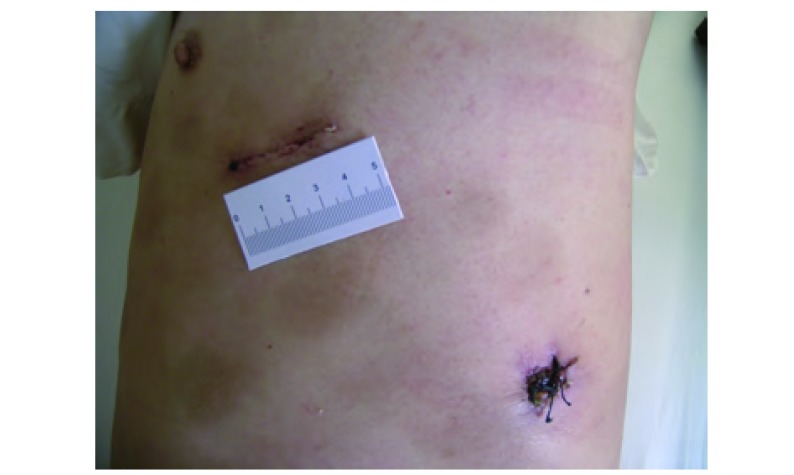
手术切口照片 The photograph of operative incision

## 2 结果

全部患者手术顺利，无中转开胸。术中出血量80 mL-300 mL，平均（110.5±24.6）mL；手术时间90 min-300min，平均（132.7±16.2）min。患者术后自觉切口疼痛较轻，很少使用镇痛剂，生活自理较早恢复且无感觉异常，肩关节活动范围与术前一样。肺癌患者淋巴结清扫范围右侧包括2、4、7、9、10、11组淋巴，左侧患者包括第5、6、7、9、10、11组淋巴结。肺癌患者每例清扫淋巴结8-12粒，平均10.2粒。术后病理检查发现淋巴结转移2例，分别是第10组、第11组。胸腔引流管拔出时间平均（3.1±1.3）d，术后平均住院时间（5.2±1.2）d。全部患者术后恢复顺利，无肺不张、肺部感染、出血等严重并发症，无围手术期死亡。

## 3 讨论

微创治疗理念已被广泛接受，并成为21世纪外科发展的重要趋势，电视腔镜技术是实现微创外科的重要途径。VATS是近年来胸外科的最大进展之一，通过专用设备借助数个小切口完成胸部手术。与常规开胸手术相比，VATS手术创伤小、疼痛轻，术后恢复快。由于不破坏胸壁的完整性，对心肺功能影响小，心肺功能差、不能耐受常规开胸手术患者也可以选择这一手术方式。

电视胸腔镜肺叶切除手术切口设计十分重要。在早期多采用McKenna教授设计4切口方法，即1个胸腔镜孔、1个主操作孔和2个副操作孔，该方法可以从各个角度解剖肺门，符合常规开胸的手术习惯，经过一段时间的探索和练习，许多医生发现减少1个副操作孔，同样可以完成VATS肺叶切除手术。目前在切口选择问题上已基本达成共识，一般取3个切口，即：①胸腔镜孔，腋中线第8肋间长约1.5 cm，置入胸腔镜，手术结束时，经此切口放置胸管；②主操作孔，腋前线和锁骨中线之间第3、4或第5肋间长约3 cm-5 cm，主要用于肺门的解剖和标本的取出；③副操作孔，腋后线与肩胛下线之间第6、7、8或第9肋间长约1. 5 cm，用于辅助操作和置入内镜切割缝合器。但副操作孔有以下缺点：背部肌肉层次多、血供丰富，易出血且不易止；胸腔较小的患者，器械进入胸腔后行程短，活动空间小，操作困难；后胸壁肋间隙窄，容易损伤肋间神经，患者术后疼痛常常来自于此切口，常有感觉异常和轻度运动障碍^[4]^。

受近年来经自然孔道和单孔道手术技术的影响，我们和许多学者一样尝试不做副操作孔，仅做一个主操作孔，以完成纵隔淋巴结活检、自发性气胸、肺楔形切除、纵隔肿瘤切除等简单VATS手术，收到很好效果^[5, 6]^。由于腋前线切口处肌肉层次少，多为肋间肌，主要是支持和保护功能，所以术后疼痛轻，对感觉和运动的影响较小。

2009年9月我们成功地实施了首例单操作孔胸腔镜肺叶切除术，至今我们共完成21例，手术过程顺利，无意外损伤和中转开胸。全组患者术后无严重心肺并发症，无围手术期死亡，患者术后切口疼痛和感觉运动异常的发生率明显降低，是一种更微创的VATS肺叶切除手术方式，具有一定的临床应用优势。我们主要有以下几点体会：①单操作孔VATS肺叶切除术的适应症与常规VATS肺叶切除相同；②胸腔镜孔后移至腋后线第8肋间，该位置与肺门连线垂直于肺门血管，经此孔放置内镜切割缝合器离断肺门大血管角度很合适；③主操作孔位置因手术方式而变化，上叶切除选第3或第4肋间，下叶切除选第5肋间，长约4 cm-5 cm。上叶肺血管切断时可将胸腔镜移至主操作孔，内镜切割缝合器经胸腔镜孔置入，行下叶切除时所有器械都经主操作孔进入；④肺门解剖方法因人而异，若肺裂较全时，按先动脉，后静脉，最后支气管的方法处理。上、中叶切除和肺裂不全者，采用先静脉，后动脉、支气管的方法处理；⑤30度镜更适合单操作孔VATS肺叶切除术，可以更好地显露术野，整个胸腔几乎无死角；⑥双腔气管插管位置合适，术侧肺完全塌陷有利于术野显露，必要时可适当调整手术床位置，利用肺叶自重显露肺门。总之，对于熟练掌握胸腔镜操作的胸外科医师，只要病例选择得当，单操作孔胸腔镜行肺叶切除是安全、可行的。

本组患者中肺癌占72.6%（16/21）。对VATS能否达到同开胸手术一样的淋巴结清扫标准一直存在争议。最近的研究^[7, 8]^表明VATS治疗早期肺癌的疗效与传统开胸手术疗效一致，甚至优于后者。对于单操作孔胸腔镜下淋巴结清扫，我们认为在30度胸腔镜下胸腔内视野几乎无死角，而且具有视觉放大作用，借助腔镜用手术器械完全可以完成同开胸手术同质量的系统性纵隔淋巴清扫。

## 编委点评

**单操作孔胸腔镜肺叶切除术**

孙玉鹗

中国人民解放军总医院胸外科

随着电视腔镜技术的迅猛发展与日臻成熟，外科已经进入了微创时代。在为患者解除病痛的同时，尽可能减轻医源性创伤始终是医学发展的主旋律。在这一理念的指导下，各种微创技术一直在不断地创新。近年来开展的经自然腔道和单孔道内镜外科手术，就是现代微创外科技术不断创新的标志。2007年法国医生完成经阴道胆囊切除术，2008年新英格兰医学杂志报道了经脐单孔肾切除术。与其它外科领域相同，普胸外科的微创手术也在不断地发展和改进。

电视胸腔镜手术（video-assisted thoracoscopic surgery, VATS）在肺癌中的应用经过诸多临床研究证实，Ⅰ期肺癌行VATS肺叶切除并淋巴结清扫是安全、可行的。基于VATS患者术后恢复快、并发症较少的优势，目前NCCN指南认为只要不违反肿瘤治疗和胸部手术原则，VATS是可手术肺癌患者合理的术式选择。随着VATS技术的不断进步，VATS已成功地用于全肺切除、支气管袖式肺叶切除等高难度手术。

VATS肺叶切除术同其它微创技术一样，也在不断地发展和改进。华西医院刘伦旭教授开展的“单向式胸腔镜肺叶切除术”就是一个例证。本期刊登的解放军总医院初向阳教授提出的“单操作孔胸腔镜肺叶切除术”是VATS技术的又一进步，该术式更加符合微创的理念。当然，这种术式的临床价值还有赖于大宗病例的长期随访，手术器械也需要进一步改进。刊出该文，是希望更多的胸外科同道尝试这一术式，不断改进和完善VATS肺叶切除手术，推动我国肺癌微创外科治疗的水平。
